# Synthesis of the Thomsen-Friedenreich-antigen (TF-antigen) and binding of Galectin-3 to TF-antigen presenting neo-glycoproteins

**DOI:** 10.1007/s10719-020-09926-y

**Published:** 2020-05-04

**Authors:** Marius Hoffmann, Marc R. Hayes, Jörg Pietruszka, Lothar Elling

**Affiliations:** 1 Laboratory for Biomaterials, Institute for Biotechnology and Helmholtz-Institute for Biomedical Engineering, RWTH Aachen University, Pauwelsstraße. 20, 52074 Aachen, Germany; 2 Institute for Bioorganic Chemistry, Heinrich Heine University Düsseldorf at Forschungszentrum Jülich, 52426 Jülich, Germany; 3 Forschungszentrum Jülich, IBG-1: Biotechnology, 52426 Jülich, Germany

**Keywords:** Thomsen-Friedenreich-antigen, Glycosynthase, Neo-glycoproteins, Multivalency, Galectin-3

## Abstract

The Thomsen-Friedenreich-antigen, Gal(β1–3)GalNAc(α1-*O*-Ser/Thr (TF-antigen), is presented on the surface of most human cancer cell types. Its interaction with galectin 1 and galectin 3 leads to tumor cell aggregation and promotes cancer metastasis and T-cell apoptosis in epithelial tissue. To further explore multivalent binding between the TF-antigen and galectin-3, the TF-antigen was enzymatically synthesized in high yields with GalNAc(α1-EG3-azide as the acceptor substrate by use of the glycosynthase BgaC/Glu233Gly. Subsequently, it was coupled to alkynyl-functionalized bovine serum albumin via a copper(I)-catalyzed alkyne-azide cycloaddition. This procedure yielded neo-glycoproteins with tunable glycan multivalency for binding studies. Glycan densities between 2 and 53 glycan residues per protein molecule were obtained by regulated alkynyl-modification of the lysine residues of BSA. The number of coupled glycans was quantified by sodium dodecyl sulfate polyacrylamide gel electrophoresis and a trinitrobenzene sulfonic acid assay. The binding efficiency of the neo-glycoproteins with human galectin-3 and the effect of multivalency was investigated and assessed using an enzyme-linked lectin assay. Immobilized neo-glycoproteins of all modification densities showed binding of Gal-3 with increasing glycan density. However, multivalent glycan presentation did not result in a higher binding affinity. In contrast, inhibition of Gal-3 binding to asialofetuin was effective. The relative inhibitory potency was increased by a factor of 142 for neo-glycoproteins displaying 10 glycans/protein in contrast to highly decorated inhibitors with only 2-fold increase. In summary, the functionality of BSA-based neo-glycoproteins presenting the TF-antigen as multivalent inhibitors for Gal-3 was demonstrated.

**Electronic supplementary material:**

The online version of this article (10.1007/s10719-020-09926-y) contains supplementary material, which is available to authorized users.

## Introduction

A carbohydrate structure of particular importance in *O*-glycosylation of mammalian glycoproteins is the Thomsen-Friedenreich-antigen (TF-antigen; Gal(β1–3)GalNAc(α1-*O*-Ser/Thr; core 1). Deriving from its precursor GalNAc(α-*O*-Ser/Thr (Tn-antigen), the TF-antigen serves as scaffold for longer and more complex mucin-type *O*-glycan structures [1]. High levels of Tn- and TF-antigen occur in 70–90% of all human carcinomas [1, 2]. They are mostly present on the cell-surface bound glycoprotein mucin-1 (MUC1) and considered as “pancarcinoma antigens” [3].

The TF-antigen has been shown to be a potent ligand in both galectin-1 (Gal-1) and galectin-3 (Gal-3) mediated cell interactions with Gal-3 being the stronger binding partner [4–8]. Gal-3 is expressed on the outer cell membrane of endothelial cells as well as carcinoma cells and binds to the TF-antigen presented on MUC1 under static and flow conditions [9]. Circulating Gal-3 was shown to bind to MUC1 on the surface of cancer cells, thereby polarizing the cell surface so that the exposure of adhesion molecules leads to cellular aggregation and tumor formation [10]. Thus, the interaction between Gal-3 and the TF-antigen plays a crucial role in homotypic aggregation of cancer cells as well as in the initial adhesion and proliferation of carcinoma cells in the vascular endothelium [2, 3, 11]. Since these mechanisms are among the first steps during cancer metastasis and proliferation, small molecule antagonists with the potential of preventing the binding of Gal-3 have been developed [12, 13]. In addition, multivalent synthetic ligands have been proven as potent binders of Gal-3 with K_D_-values in the low nanomolar range [14–16]. Especially, multivalent neo-glycoproteins (NGPs) on the basis of bovine serum albumin (BSA) displaying the LacdiNAc-LacNAc motif, GalNAc(β1–4)GlcNAc(β1–3)Gal(β-GlcNAc, or thiodigalactosides were demonstrated as effective ligands for Gal-3 with sub-nanomolar K_D_-values [17–19]. The presentation of a single TF-antigen ligand on synthetic glycopeptides was favorable to gain affinity for Gal-3 binding when compared to a non-conjugated TF-antigen and glycomimetics [20, 21]. Moreover, multivalent mucin-like presentation of the TF-antigen triggers Gal-3 binding as demonstrated for a TF-antigen presenting anti-freeze protein from codfish, which suppresses prostate cancer metastasis and T-cell apoptosis in mice [22].

However, so far and to the best of our knowledge, the effect of multivalent presentation of the TF-antigen on BSA-based NGPs for affinity studies with Gal-3 has not been investigated yet.

**Scheme 1:**
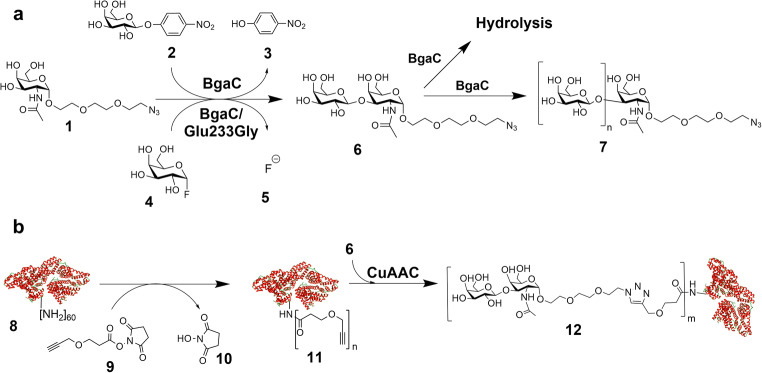
**a:** Synthesis of the TF-antigen-azide **6** with BgaC and the glycosynthase mutant BgaC/Glu233Gly. **b:** Synthesis of alkynyl-functionalized BSA and coupling of **6** by *click* chemistry. The maximum theoretical number of available amines (*n* = 60) is based on the sum of ε-amino groups of lysines in the BSA sequence (59) and the N-terminus of the protein (1). m: range of coupled glycans/BSA. BSA protein structure was modified from PDB entry 3 V03 (rcsb.org) [33] using UCSF Chimera [34]. **1**: GalNAc(α1-EG3-azide; **2**: *p*-nitrophenyl-β-d-galactoside (*p*NP-Gal); **3**: *p*-nitrophenol (*p*NP); **4**: α-d-galactopyranosyl fluoride (α-GalF); **5**: Fluoride; **6**: Gal(β1–3)GalNAc(α1-EG3-azide; **7**: Galactosyl-oligosaccharide (GAOS); **8**: Bovine serum albumin (BSA); **9**: Propargyl-EG1-NHS-ester; **10**: *N*-hydroxy-succinimide; **11**: Alkynyl-functionalized BSA; **12**: TF-antigen-presenting neoglycoprotein.

**Scheme 2:**
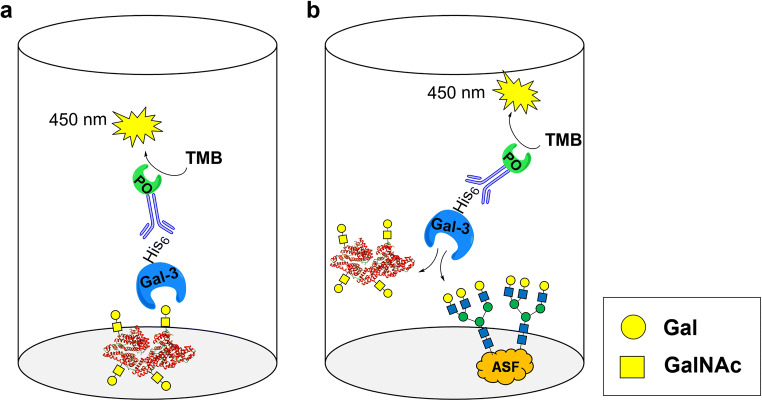
Representation of the two types of ELLAs used in this study. **a** NGPs with different glycan densities are immobilized in the well of a microtiter plate. Binding of Gal-3 is quantified via the conversion of 3,3′,5,5′-tetramethylbenzidine (TMB) by a peroxidase (PO) coupled to an anti-His_6_-antibody. **b** NGPs are used as inhibitors in solution for the binding of Gal-3 to immobilized asialofetuin (ASF)

We herein report on the synthesis and tunable multivalent presentation of Gal(β1,3)GalNAc(α1-EG3-azide (TF-antigen-azide) on BSA-based NGPs and their binding interaction with Gal-3 (Scheme 1). We compared the glycosidase BgaC from *Bacillus circulans* and its glycosynthase mutant BgaC/Glu233Gly [23] regarding the synthesis of the TF-antigen-azide, which we subsequently coupled to alkynyl-functionalized lysine residues of BSA via a copper(I)-catalyzed alkyne-azide cycloaddition (CuAAC). Varying glycan densities between 2 and 53 glycans per BSA were obtained and Gal-3 binding properties of the multivalent NGPs were evaluated by an enzyme-linked lectin assay (ELLA) (Scheme 2).

## Materials and methods

### Materials

GalNAc(α1-(tri(ethylene glycol))-azide (GalNAc(α1-EG3-azide, **1**) was purchased from Sigma Aldrich (now Merck, Darmstadt, Germany). Tris(3-hydroxypropyltriazolylmethyl) amine (THPTA) for CuAAC was obtained from TCI Chemicals (Eschborn, Germany). Propargyl-EG1-NHS (**9**) ester was obtained from Broadpharm (San Diego, USA). *p*-nitrophenyl β-d-galactopyranoside (*p*NP-Gal, **2**) was purchased from Carbosynth (Berkshire, United Kingdom). All other chemicals, if not specifically mentioned, were obtained from Carl Roth (Karlsruhe, Germany).

### Synthesis of α-GalF (**4**)

The synthesis of the glycosyl donor α-d-glucopyranosyl fluoride (α-GalF; **4**) was carried out as previously described by Henze et al. [24]. The peracetylated galactose (5 mmol, 2 g) was dissolved at 0 °C in HF/pyridine solution (1 mL/mmol, 70% HF in pyridine). The reaction was stirred and monitored by TLC. After completion, the reaction was diluted with CH_2_Cl_2_ and dH_2_O. Excess fluoric acid was neutralized using K_2_CO_3_ and the organic phase separated from the aqueous. Pyridine was removed by extraction with saturated CuSO_4_-solution and the organic phase dried over MgSO_4_ and filtered. The solvent was removed under reduced pressure and the product mixture purified by flash chromatography (45% α-anomer, 2.3 mmol, 0.8 g). Deacetylation occurred with 2 m ammonia in methanol at 0 °C. After complete conversion the solvent was evaporated under reduced pressure. α-GalF **4** was obtained without further purification as colorless solid (quant., 0.4 g).

### Expression and purification of recombinant enzymes and galectin-3

The recombinant galactosidase and galactosynthase were expressed and purified as described before [24, 25]. In brief, *E. coli* BL21 (DE3) cells were transformed with the plasmid pETDuet-1 carrying the recombinant gene for the galactosidase BgaC or the galactosynthase BgaC/Glu233Gly, respectively. Expression of the recombinant gene was conducted for 24 h in TB-medium at 25 °C after induction with 0.1 mM IPTG. Purification of both enzymes was achieved by immobilized metal-ion chromatography (IMAC).

Recombinant Gal-3 protein was produced and purified as described previously [26]. Human Gal-3 was expressed with an N-terminal His_6_-Tag in *E. coli* Rosetta (DE3) pLysS and purified via IMAC. After purification, the lectin was stored at 4 °C in phosphate buffered saline (PBS) containing 2 mM EDTA.

### Enzymatic activity of BgaC β-galactosidase

Hydrolytic activity of the BgaC galactosidase was determined as described previously [25]. 100 μL of appropriately diluted enzyme solution were added to 900 μL of 4.4 mM **2** in 50 mM citrate-Na_2_HPO_4_buffer (pH 6.0) and incubated for 5 min. Samples of 100 μL were taken at different time points and stopped by the addition of 200 μL of 200 mM Na_2_CO_3_. Subsequently, the signal was measured at 405 nm. Quantification was done via a *p*-nitrophenol (*p*NP, **3**) standard curve.

### Enzymatic activity and kinetic analysis of BgaC/Glu233Gly galactosynthase

Enzymatic activity of the galactosynthase BgaC/Glu233Gly was determined via a discontinuous HPLC assay. 10 mm**4** and 5 mm of **1** were added to 50 mm HEPES buffer (pH 7.2) and pre-heated to 30 °C. Enzyme solution containing 600 μg of protein was added to start the reaction. The total volume was 100 μL. Samples of 10 μL were inactivated at 95 °C for 5 min, centrifuged and diluted with water (1:5). Reversed-phase HPLC was conducted on a Multochrom 100–5 C18 column (250 × 4 mm, CS Chromatographie, Langerwehe, Germany) with an isocratic eluent consisting of 90% water and 10% acetonitrile and a flow rate of 1 mL/min. One unit of enzyme activity is defined as the amount of enzyme that converts 1 μmol acceptor substrate per minute.

For acceptor substrate kinetics, the concentration of **1** was varied between 0 and 55 mm while the donor substrate **4** was fixed at 25 mm. For the donor substrate kinetics, **4** was varied while the acceptor substrate **1** was fixed at 5 mm. Values for K_M_ and v_max_ were calculated using SigmaPlot.

### Synthesis of Gal(β1,3)GalNAc(α1-EG3-azide (**6**) using BgaC galactosidase

Synthesis was conducted on a 1 mL scale. The solution contained 10 mm**2** as well as 5 mm**1** in 50 mm Na_2_HPO_4_-citrate buffer (pH 6.0). The final concentration of the enzyme was 0.3 U/mL. Samples were taken at different time points and the reaction was stopped at 95 °C for 5 min. After centrifugation and appropriate dilution, the supernatant was analyzed via HPLC. The flow rate was 1 mL/min. The ratio of the two solvents in the eluate at given time points was as follows: 90% H_2_O for 15 min, decrease to 40% H_2_O within 5 min, steady 40% H_2_O for 5 min, return to 90% H_2_O within 2 min. Peaks were detected at 205 nm and quantification was carried out via external calibration curves.

### Synthesis and purification of Gal(β1,3)GalNAc(α1-EG3-azide (**6**) using galactosynthase BgaC/Glu233Gly

Semi-preparative synthesis was performed in a volume of 2 mL containing 10 mm**1** and 20 mm**2** in 50 mm HEPES buffer (pH 7.2). The synthesis was monitored via HPLC as described above. After the reaction was finished, the mixture was heated to 95 °C for 5 min and subsequently centrifuged. Remaining enzyme in the supernatant was removed by ultracentrifugation (Vivaspin® (Sartorius), 30 kDa MWCO) and the volume was reduced via vacuum evaporation (Eppendorf concentrator plus, Eppendorf, Germany). The product **6** was isolated via preparative HPLC (Knauer Smartline) with column Multokrom 100–5 C18 (250 × 20 mm) by applying an isocratic eluent consisting of 90% water and 10% acetonitrile and a flow rate of 12.5 mL/min. The product fractions were collected and the solvent was evaporated to dryness. The final product was used for further analysis and subsequently dissolved in water.

### Mass spectrometry

Analysis of the molecular mass of the reaction products was carried out via ESI-MS (Finnigan Surveyor MSQ Plus, Thermo Scientific, needle voltage = 4 kV, temperature = 400 °C, cone voltage = 100 V, negative mode) via LC-MS-measurement.

### Nuclear magnetic resonance

NMR spectra were recorded on Bruker Avance/DRX600 spectrometer (^1^H: 600 MHz, ^13^C: 151 MHz) in D_2_O and CDCl_3_ at 30 °C. The residual solvent signals were used as references (CDCl_3_: δ_H_ 7.26 ppm, δ_C_ 77.16 ppm; D_2_O: δ_H_ 4.79 ppm). Chemical shifts (δ) were reported in parts per million (ppm) and the coupling constants (*J*) in Hertz (Hz). The anomeric configuration of the glycosyl moieties was determined by the coupling constant (*J*) between the anomeric 1-H and the 2-H. The linkage of the glycosidic bond was determined by coupling signals between the 3′-C and 1″-C observed via ^1^H-^13^C-HMBC NMR analysis.

The product of the galactosynthase reaction was dissolved in D_2_O and analyzed via ^1^H-NMR spectroscopy. Due to the low resolution and high overlap of the peaks in the acquired spectrum, the product was peracetylated and analyzed via ^1^H- and ^13^C-NMR using CDCl_3_ as the solvent (Fig. S8-S9). Peracetylation was carried out as described by Steinmann et al. [26]. Briefly, the enzymatic product was dissolved in pyridine (1 mL/mmol), acetic anhydride (1.5 eq/OH-group) added, and stirred over night at 25 °C. The reaction was diluted with ethyl acetate and pyridine removed by extraction with saturated CuSO_4_-solution. The organic phase was dried over MgSO_4_ and subsequently filtered. The solvent was then evaporated under reduced pressure.

### Alkynyl-modification of BSA (**8**)

Prior to modification, BSA **8** was delipidated via activate charcoal treatment as described previously [27]. Depending on the desired degree of alkynyl-modification, 16.7 μm delipidated BSA was incubated with 0.2–20-fold molar excesses of Propargyl-EG1-NHS-ester **9** (Broadpharm, San Diego, USA) for 1.5 h in 50 mm PBS (pH 7.4) at 25 °C. NHS **10** and unreacted linker were removed via ultracentrifugation (Vivaspin® 500 (Sartorius), 10,000 Da MWCO). The degree of alkynyl-modification of **11** was determined via SDS-PAGE (8% polyacrylamide gels, 25 mA constant current) and the trinitrobenzene sulfonic acid (TNBSA)-assay as reported previously [28]. Briefly, 50 μL of 12.5 μM **11** in sodium tetraborate buffer (pH 9.0) were added to 50 μL of 7.5 mM TNBSA in the same buffer. The mixture was incubated for 15 min at room temperature. Subsequently, the absorbance was measured at 420 nm. Each sample was analyzed in triplicates and the standard deviation was calculated. Quantification was achieved by comparing the signal of alkynyl-modified BSA to unmodified BSA.

### Coupling of TF-antigen-azide (**6**) to BSA-alkynyl proteins (**11**) via CuAAC

Conjugation of glycan molecules with **11** was carried out via CuAAC based on protocols described elsewhere [29, 30]. The final concentrations of reagents (except for the buffer) varied with the degree of alkynyl-functionalization based on the results of the TNBSA-assay. However, the molar ratios of the substances were unchanged in the respective reactions and amounted to Gal(β1,3)GalNAc(α1-EG3-azide **6**/alkynyl groups/Cu(I)/THPTA/ascorbate (1/0.5/2/10/40). For example, 1 mm of **6** was added to 0.5 mm alkynyl residues present on **11** in K_2_HPO_4_-buffer (final concentration 100 mm, pH 7.2). A premixed solution of CuSO_4_ and THPTA was added to the solution to a final concentration of 2 mm and 10 mm, respectively. The addition of sodium ascorbate (final concentration 40 mm) initiated the reaction. The mixture was incubated for 3 h at 37 °C. For use in further experiments, neo-glycoproteins **12** were purified from the mixture via ultracentrifugation (Vivaspin® 500, 10,000 Da MWCO). The coupling of glycan molecules was assessed and quantified via SDS-PAGE (8% polyacrylamide gels, 25 mA constant current).

### Enzyme-linked Lectin assay (ELLA)

For the binding assay (Scheme 2a), the respective neo-glycoprotein **12** (50 μL, 0.1 μm) was immobilized overnight in triplicates in wells of a 96-well microplate (MaxiSorp, Nunc, Wiesbaden, Germany). 200 μL of 2% [*w*/*v*] BSA in PBS was added and incubated for 1 h to block residual binding sites. Subsequently, 50 μL of Gal-3 in PBS supplemented with 2 mm EDTA was added to each well in varying concentrations and incubated for 1 h. Anti-His_6_-Peroxidase (Roche, Mannheim, Germany) was added for 1 h to detect bound His_6_-tagged galectin. Quantification was achieved via the conversion of 50 μL TMB One substrate by the immobilized peroxidase to a blue solution. The reaction was stopped after 45 s by the addition of 50 μL of 3 m HCl to yield a yellow solution. The signal was read out at 450 nm. Between the steps, wells were washed three times with 200 μL PBS supplemented with 0.05% [*v*/*v*] Tween-20 (PBS-T).

For the competitive inhibition assay (Scheme 2b), 50 μL of 0.1 μm (5 pmol) asialofetuin (ASF) per well were incubated in triplicates overnight in the wells of a microplate (MaxiSorp, Nunc, Wiesbaden, Germany). Subsequently, 200 μL of 2% [*w*/*v*] BSA were added and incubated for 1 h to prevent unspecific adsorption of proteins. 25 μL of inhibitor (lactose, TF-antigen-azide **6** or NGP **12**) in various concentrations was added in triplicates, directly followed by 25 μL of Gal-3 (25 μm final concentration). Samples were incubated for 1 h. Signal detection was conducted as mentioned above for the binding assay. Between each step, wells were washed three consecutive times with 200 μL PBS-T.

## Results

### Enzymatic synthesis of TF-antigen-EG3-azide (**6**)

In our previous studies, we reported on the generation of the galactosynthase BgaC/Glu233Gly by rational design based on the crystal structure of BgaC from *B. circulans* and its use in combination with glycosyltransferases for the synthesis of various type 1 and type 2 poly-LacNAc structures [23, 24]. The same enzyme variant has been shown to catalyze the formation of 4-nitrophenyl α-d-2-*N-*galacto-*N-*biose (*p*NP-αGNB, Gal(β1,3)GalNAc(α-*p*NP) using 4-nitrophenyl α-d-2-*N*-acetylgalactosaminide (*p*NP-αGalNAc) as the acceptor substrate [31]. The kinetic analysis in this study suggested that the galactosynthase prefers α-configured *p*NP-HexNAc-glycosides over β-configured glycosides as acceptor substrates.

**Fig. 1:**
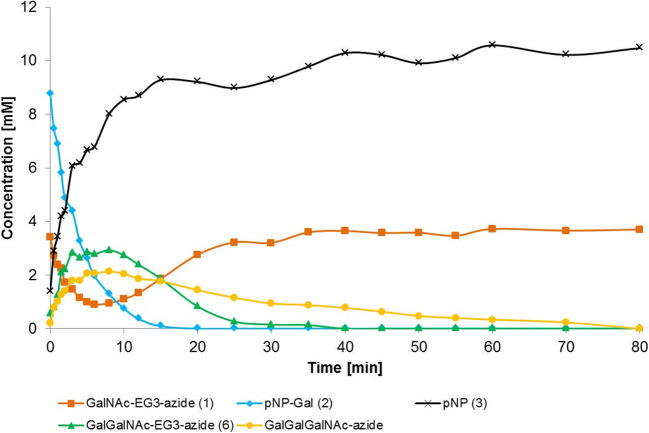
Synthesis of the TF-antigen-EG3-azide **6** with BgaC (Scheme 1a). The synthesis reaction was conducted with 10 mM *p*NP-Gal **2** and 5 mM GalNAc(α1-EG3-azide **1** in 50 mM Na_2_HPO_4_-citrate-buffer (pH 6.0). *p*NP-Gal **2** was used as the donor substrate. *p*NP **3** is a side product of the reaction. Analysis was carried out via HPLC at a detection wavelength of 205 nm. Quantification was done by external standard curves

In the present study, we first compared the synthesis of the TF-antigen-EG3-azide **6** with the wild type galactosidase enzyme BgaC and the galactosynthase BgaC/Glu233Gly, respectively, using the novel acceptor substrate GalNAc(α1-EG3-azide **1** (Scheme 1A). Reactions with BgaC resulted in a low synthetic yield (approx. 60% after 10 min reaction time) with concomitant formation of a trisaccharide product as well as galacto-oligosaccharides (GAOS, 7) (Fig. 1 and Fig. S1 in supporting information). The identity of the side products was proven by analysis of the respective peak masses by LC-ESI-MS (Fig. S2-S4). After a reaction time of 10 min, the concentration of product **6** decreased while concentration of the acceptor substrate **1** increased again until the initial reaction conditions concerning these compounds were restored. The donor substrate *p*NP-Gal was depleted after approx. 15 min.

**Fig. 2:**
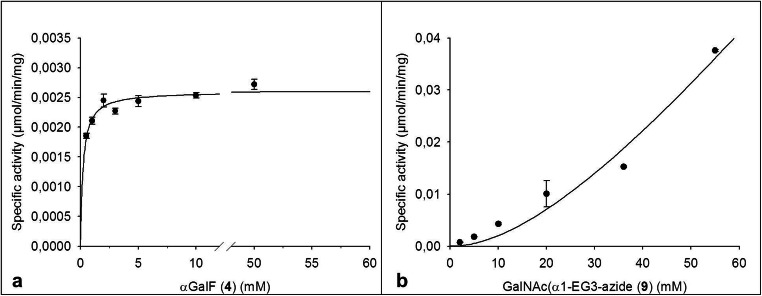
Kinetic analysis of BgaC/Glu233Gly regarding the substrates α-GalF **4** (a) and GalNAc(α1-EG3-azide **1** (b). (a) was fitted according to the Michaelis-Menten equation (Eq. S1). Vmax: Not determined; K_M_: Not determined. (b) was fitted according to the Hill equation (Eq. S2). V_max_: 2.6 mU/mg; K_M_: 0.22 M. Each data point was generated in triplicates. The standard deviation of the mean is provided in the form of error bars

Glycosynthases are known for their lack of hydrolytic activity by replacing the nucleophilic amino acid in the reactive center by a small non-nucleophilic residue [32]. We concluded that BgaC/Glu233Gly is a suitable tool for the synthesis of the TF-antigen-EG3-azide **3.** Kinetic analysis revealed an apparent K_M,app_ of 0.22 mm and a v_max_ of 2.6 mU/mg for the donor substrate **4** (Fig. 2). Regarding the acceptor substrate **1**, saturation of the fitted curve was not obtained with reasonable substrate concentrations. Consequently, K_M_ and v_max_ were not determined in this study.

**Fig. 3:**
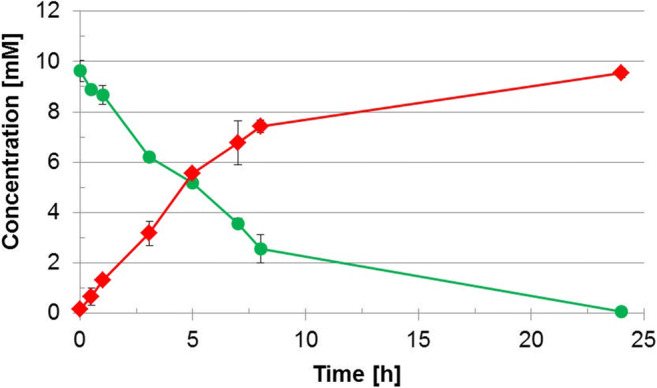
Synthesis of the TF-antigen-azide **6** by BgaC/Glu233Gly (Scheme 1a). Conversion of GalNAc(α1-EG3-azide **1** () to the TF-antigen-azide **6** (). The synthesis reaction was conducted in triplicates on a 2 mL-scale with 20 mm οf **4** and 10 mm of **1** in 50 mm Na_2_HPO_4_-citrate-buffer (pH 6.0). Standard deviations of the mean are provided as error bars

Galactosynthase BgaC/Glu233Gly was applied in the synthesis of the TF-antigen-EG3-azide **6** (Scheme 1A) resulting in 97% conversion of **1** after 24 h (Fig. 3). The formation of a single product peak was observed by HPLC (Fig. S5). The product peak was isolated by preparative RP-HPLC with a final yield of 80% [mol/mol]. Analysis by LC-ESI-MS confirmed the calculated corresponding molecular ion mass of 539.3 g/mol for **6** (Fig. S6). The β1,3-linkage of **6** was verified via ^1^H-NMR and ^13^C-NMR (Fig. S7-S9).

### Synthesis and analysis of TF-antigen-neo-glycoproteins (**12**)

**Fig. 4:**
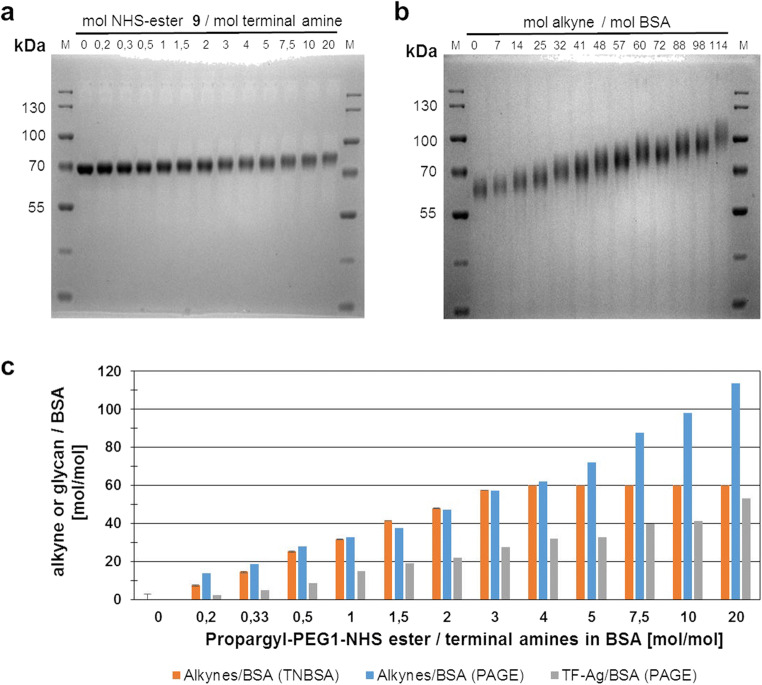
Results of the alkynyl-functionalization of amine residues of BSA and subsequent coupling of TF-antigen-EG3-azide **6** to alkynyl-modified BSA **11** via CuAAC chemistry (Scheme 1B). M: PageRuler Prestained Protein Ladder. Each gel was loaded with 2 μg/lane of protein. **a** SDS-PAGE of BSA samples after alkynyl-functionalization with the indicated molar ratios of propargyl-EG1-NHS ester **9** and terminal amine residues. The number of terminal amines consists of the number of ε-amines from lysine residues (59) plus the N-terminus of the protein (1). The increase in molecular weight (MW) compared to unmodified BSA is 0.111 kDa per coupled propargyl-EG1-moiety. **b** SDS-PAGE of neo-glycoproteins after CuAAC-catalyzed coupling of TF-antigen-EG3-azide **6** to the alkynyl-modified BSA samples. The increase in MW compared to unmodified BSA is 0.652 kDa per coupled glycan. **c** Comparison of the number of alkynyl residues derived from the TNBSA assay and SDS-PAGE and number of TF-antigens per BSA according to SDS-PAGE analysis. The retardation factor (R_f_) of the samples in the gel was determined and their molecular weight was calculated by linear regression based on the protein size standard (lane M) (Fig. S10). The number of attached propargyl-EG1-linker and glycans was calculated by the differences in molecular weights between unmodified BSA **8** and modified BSA samples **11** and **12**, respectively. The TNBSA assay was conducted in triplicates. Standard deviations of the mean are provided as error bars

The purified product **6** was used for the synthesis of BSA-based neo-glycoproteins (NGPs **12**) (Scheme 1B). First, alkynyl-functionalization of the ε-amino groups of lysines of BSA **8** by propargyl-EG1-NHS ester **9** was performed. BSA **8** consists of 583 amino acids with 59 incorporated lysine residues (UniProt accession number **P02769**). Including the N-terminus of the protein, this sums up to 60 potential modification sites. The degree of BSA alkynyl-functionalization was controlled by varying the molar ratio between **9** and the amine groups. The number of modified sites in **11** was determined by quantification of the remaining unmodified lysine residues via a TNBSA assay before and after treatment with **9**. In addition, reducing SDS-PAGE was performed to further assess the degree of alkynyl-modification. Subsequent coupling via CuAAC with a two-fold molar amount of TF-antigen-EG3-azide **6** over the alkynyl-labeled amino residues resulted in NGPs with a varying number of coupled glycans. Molecular weight shifts of NGPs were determined by the difference in electrophoretic mobility before and after coupling (Fig. 4, Table 1 and Fig. S10).

**Table 1:** Number of alkynylated and glycosylated sites in the BSA protein sequence according to analysis via TNBSA assay and SDS-PAGE

Propargyl-EG1-NHS-ester 9/ terminal amine [mol/mol]	Alkynes/BSA (TNBSA) [mol/mol]	Alkynes/BSA (PAGE) [mol/mol]	TF-Ag/BSA (PAGE) [mol/mol]	Yield CuAAC [%]
0	0 ± 1.4	0	0	–
0.2	7 ± 0.5	14	2	32
0.33	14 ± 1.3	19	5	33
0.5	25 ± 0.9	28	9	34
1	32 ± 0.6	33	15	47
1.5	41 ± 0.1	38	19	46
2	48 ± 0.5	47	22	45
3	57 ± 0.0	57	28	48
4	60 ± 0.0	62	32	51
5	60 ± 0.0	72	33	45
7.5	60 ± 0.0	88	40	45
10	60 ± 0.2	98	41	42
20	60 ± 0.1	114	53	47

Yields of the *click* reaction were calculated on the basis of the number of alkynes present according to TNBSA assay (for NHS-ester **9** molar excess <4) or SDS PAGE (for NHS-ester **9** molar excess ≥4). The TNBSA-assay was conducted in triplicates. The standard deviation of the mean is provided behind the calculated number of alkynyl groups

Figure 4a indicates that an increasing excess of **9** during the coupling reaction leads to an increasing molecular weight of alkynyl-modified BSA **11**. This is also confirmed by the TNBSA assay (Fig. 4c and Table 1). The numbers of alkynyl-modified sites derived from both analytical methods are in accordance when lower molar excesses of **9** are applied. However, values vary significantly for samples treated with more than a 4-fold molar excess of **9**. The TNBSA assay shows that the maximum of 60 sites per BSA molecule carry the PEG-alkynyl moiety at a 4-fold molar excess of the linker (Fig. 4c). This number does not increase when higher amounts are used. However, SDS-PAGE analysis shows an alkynyl-modification density of up to 114 alkynyl residues per BSA molecule when the molar excess of **9** is increased to 20 (Table 1).

In a second step, the purified TF-antigen-azide disaccharide **6** was coupled to **11** via CuAAC chemistry (Scheme 1B). A molar ratio of 2:1 for azide and alkyne functional groups was applied in each reaction. The number of alkynyl-carrying residues used for the calculations was derived from the TNBSA assay for molar excess ratios of **9** below 4:1 and from SDS-PAGE for molar excess ratios above 4:1. SDS-PAGE analysis (Fig. 4c and Table 1) of NGPs **12** indicated that the mass difference before and after CuAAC in comparison to unmodified BSA increases with increasing alkynyl modification of **11**. Molecular weight shifts were calculated using linear regression (Fig. S10). Variable glycan densities between 2 and 53 glycans per BSA molecule were obtained (Table 1).

### Galectin-3 binding to immobilized TF-antigen neo-glycoproteins (**12**)

**Fig. 5:**
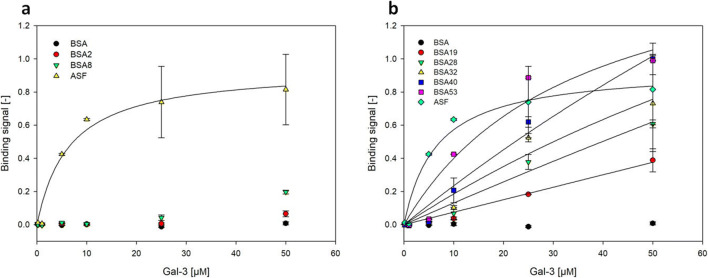
Analysis of Gal-3 binding to immobilized TF-antigen NGPs with glycan densities between 0 and 53 mol glycan / mol BSA in an enzyme-linked lectin assay (ELLA). **a:** glycan densities between 2 and 8 mol TF-antigen / mol BSA. **b:** glycan densities between 19 and 53 mol TF-antigen / mol BSA. Sample designation indicates mol TF-antigen / mol BSA. NGPs were immobilized in wells of a microplate (5 pmol/well) and incubated with varying amounts of recombinant human Gal-3. Each sample was measured in triplicates. ASF served as a positive control. Background for blank samples (no Gal-3) were subtracted from the binding signals. Final binding signal values are plotted for varying Gal-3 concentrations. All Gal-3 concentrations were analyzed in triplicates. All curves were fitted using the software SigmaPlot

Selected NGPs were immobilized in the wells of microplates for determination of the binding affinity of human galectin-3 (Gal-3) in an enzyme-linked lectin assay (ELLA) (Scheme 2 and Fig. 5). The increase of binding signals resulting from the binding of Gal-3 to immobilized NGPs with increasing glycan densities while there is no binding signal for unmodified BSA. NGPs with valencies below 8 glycans/BSA showed very weak binding signals (Fig. 5a).

However, increased binding of Gal-3 was observable to NGPs with higher glycan densities starting from ≥8 glycans (Fig. 5b). Distinguishable binding signals were observed for a Gal-3 concentration of ≥10 μm. The results confirm the accessibility of the TF-antigen ligand on the immobilized NGPs for Gal-3 binding. However, apart from ASF as a standard glycoprotein for Gal-3 binding, binding curves for the applied Gal-3 concentrations did not reach saturation. Therefore, apparent K_D_-values were not calculated.

**Fig. 6:**
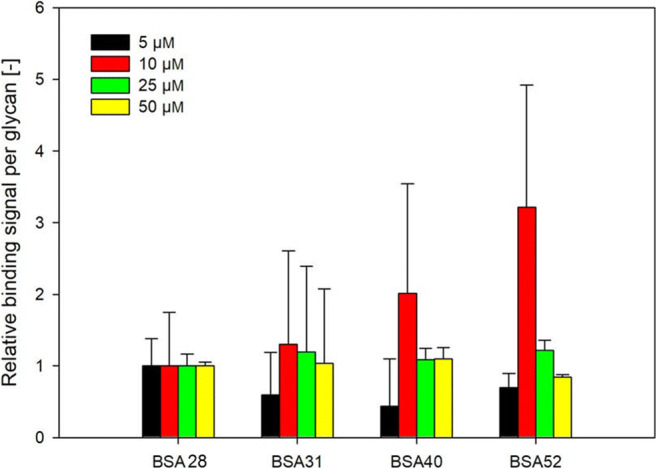
Relative binding signal per glycan for neo-glycoproteins with different glycan densities at Gal-3 concentrations between 5 and 50 μm

Furthermore, potential effects of multivalent glycan display were investigated. For this purpose, binding signals per coupled glycan were determined at different Gal-3 concentrations. For samples below 28 glycans/BSA, binding signals were not observed at Gal-3 concentrations of 5 μm or lower (Fig. 5). Therefore, NGPs with glycan densities higher than 28 glycans/BSA were compared in relation to the binding signal of BSA28 (Fig. 6).

An increase of binding signals per glycan was observed for a Gal-3 concentration of 10 μM, however, these numbers also display high standard deviations. For other Gal-3 concentrations binding signals reached approximately the same signal level per glycan. We concluded that the NGPs show no significant multivalency effect.

### Competitive inhibition of Galectin-3 binding by TF-antigen neo-glycoproteins (**12**)

**Fig. 7:**
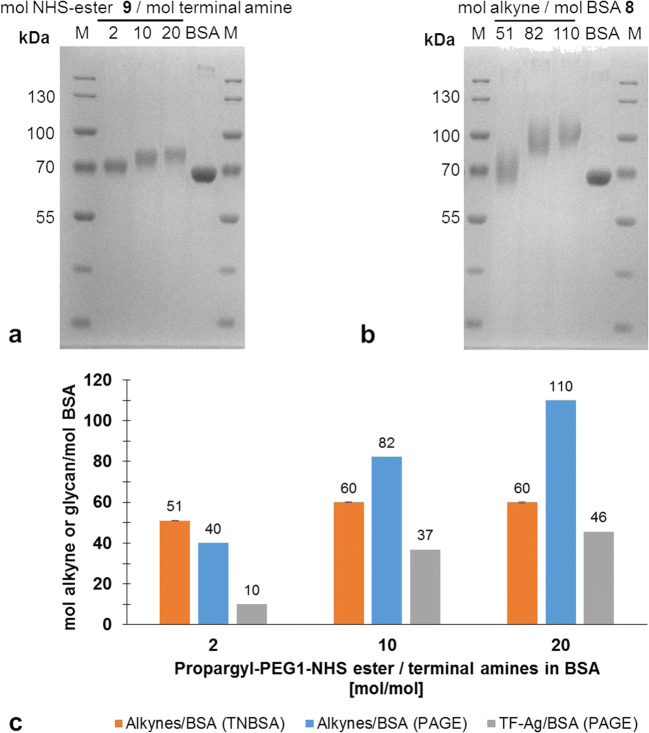
Results of the alkynyl-modification of terminal amine residues of BSA **8** and subsequent coupling of TF-antigen-azide **6** to the acylated sites via CuAAC chemistry on a 6 mL-scale. **a** SDS-PAGE of **11** after alkynyl modification with the indicated molar ratios between Proparyl-EG1-NHS ester **9** and terminal amine residues. The number of terminal amines is put together by the number of ε-amines from lysine residues (59) plus the N-terminus of the protein (1). The added molecular weight (MW) to unmodified BSA of the coupled propargyl-EG1-moiety after cleavage of the NHS-group **10** is 0.111 kDa. Each lane was loaded with 2 μg of protein. M: PageRuler Prestained Protein Ladder (ThermoFisher, Waltham, USA). **b** SDS-PAGE of NGPs **12** after CuAAC-catalyzed coupling of TF-antigen-azide **6** to **11**. The added MW to unmodified BSA of the coupled glycan is 0.652 kDa. Each lane was loaded with 2 μg of protein. M: PageRuler Prestained Protein Ladder (ThermoFisher, Waltham, USA). **c** Comparison of the number of alkynyl functionalized residues derived from the TNBSA assay and SDS-PAGE and number of TF-antigen per BSA according to SDS-PAGE analysis. The retardation factor (R_f_) of the samples in the gel was determined and their molecular weight was calculated by linear regression based on the protein size standard (lane M) (Fig. S11). The number of attached propargyl-EG1-linker and glycans was calculated by the differences in molecular weights between unmodified BSA **8** and modified BSA samples **11** and **12**, respectively, and is noted above the bars. The TNBSA-assay was conducted in triplicates

The potential of monovalent TF-disaccharide **6** as well as TF-antigen NGPs **12** to inhibit the Gal-3 interaction with ASF was investigated. In order to provide enough sample material, synthesis of NGPs **12** was performed on a larger scale (6 mL) with the aim to synthesize samples with low, medium and high glycan densities (Fig. 7 and Table S1). For this purpose, alkynyl functionalities were introduced by using 2-, 10- and 20-fold molar excesses of **9** during the coupling reaction. The resulting alkynyl modification degrees of 10, 37 and 46 glycans per BSA were comparable to the previous results on a smaller scale (Fig. 4 and Table 1). Again, the available number of alkynyl residues defined the final number of glycan residues per BSA molecule after coupling of **9** to BSA. Yields regarding CuAAC ranged between 42 and 51%.

**Fig. 8:**
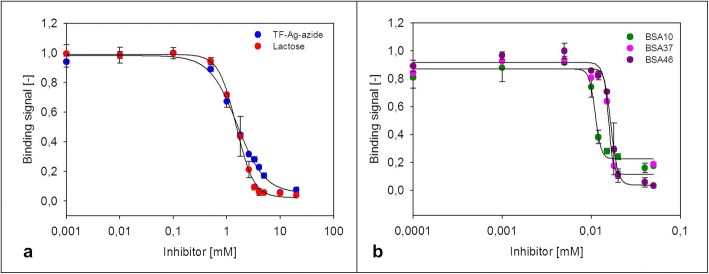
Competitive inhibition assays of Gal-3 binding to ASF by monovalent TF-antigen-azide **3** and lactose (**a**) and BSA-based neo-glycoproteins decorated with 10, 37 and 46 TF-antigen/BSA (**b**). Binding signals were detected using enzyme-linked lectin assay (ELLA). The residual binding of 25 μm Gal-3 to 5 pmol/well of immobilized ASF after incubation with varying concentrations of inhibitor is depicted. All inhibitor concentrations were analyzed in triplicates

The resulting neo-glycoproteins presenting 10 (BSA10), 37 (BSA37) and 46 (BSA46) mol TF-antigen/mol BSA, respectively, were evaluated as competitive inhibitors for Gal-3 binding to ASF using ELLA analysis (Fig. 8 and Table 2).

**Table 2:** Inhibition properties of TF-antigen-azide **3** and respective neo-glycoproteins displaying different glycan densities

Ligand	m^a^	IC_50_ [mm]	RIP^b^	RIP/Glycan^c^
Lactose	1	1.51 ± 0.0584	1	1
TF-antigen-azide (3)	1	1.56 ± 0.0926	1	1
BSA10	10	0.011 ± 0.003	142	14.2
BSA37	37	0.016 ± 0.0004	98	2.6
BSA46	46	0.017 ± 0.0004	92	2

Lactose was utilized as a positive control

^a^m: Average number of glycans per ligand molecule; ^**b**^ RIP: Relative inhibitory potency compared to TF-antigen-azide, i.e. IC_50_(unconjugated glycan)/IC_50_(neo-glycoprotein); ^**c**^ RIP/Glycan: Relative inhibitory potency per glycan compared to TF-antigen-azide, i.e. RIP/m

The monovalent disaccharides showed comparable IC_50_-values in the mm-range with 1.51 mm and 1.56 mm for lactose and TF-antigen-azide **6**, respectively. Therefore, a high concentration between 100 μm – 10 mm must be applied to inhibit the interaction between Gal-3 and the multivalent glycoprotein ASF. However, significantly lower IC_50_-values between 11 and 17 μm are observed for all tested neo-glycoproteins (Table 2). The value of relative inhibitory potency (RIP) is a benchmark for the relative strength of an inhibitor. The number of displayed TF-antigen glycans can be reduced to 10 glycans/mol BSA, which results in the highest RIP for BSA10.

The RIP per glycan quantifies the effect of multivalency on the affinity of Gal-3 towards the inhibitor. Here, BSA10 also shows the highest number and therefore the greatest multivalent effect.

## Discussion

### Synthesis of TF-antigen-EG3-azide (**6**)

A comparison was made between synthesis of the TF-antigen using the galactosidase BgaC and its glycosynthase equivalent BgaC/Glu233Gly. Both enzymes were capable of product formation with conversions of approx. 60% after 10 min and 97% after 24 h for BgaC and BgaC/Glu233Gly, respectively. BgaC shows significant product hydrolysis and formation of side products (Fig. 1) compared to the galactosynthase (Fig. 2). The transglycosylation product TF-antigen-EG3-azide **6** is hydrolyzed and also utilized as an acceptor substrate for further galactosylation yielding a trisaccharide product and further galacto-oligosaccharides with additonal galactose moieties (Scheme 1). Analysis via ESI-MS revealed the formation of **6** (Fig. S2) and the trisaccharide bearing an additional galactose (GalβGal(β1,3)GalNAc(α1-EG3-azide) (Fig. S4 and S5). In contrast to previous reports, the formation of β1,6- or β1,4-linked disaccharides resulting in distinct peaks can be excluded by LC-ESI-MS analysis as none of the observed peaks except for peak 3 revealed the mass of a Gal-GalNAc-EG3-azide disaccharide [35, 36].

In contrast, the reaction of the galactosynthase shows the formation of **6** as single product. The high product yield results from a lack in product hydrolysis [32]. In addition, BgaC/Glu233Gly, unlike other reported glycosynthase mutants [37], does not accept β1,3-linked galactosides as acceptor substrates [31]. Further reduction in yield due to the formation of GAOS is therefore prevented. Thus, application of the BgaC/Glu233Gly galactosynthase in the TF-antigen synthesis resulted in a significant increase in product yield simplifying product isolation.

We conclude that the reaction of BgaC is difficult to control and BgaC/Glu233Gly is the preferred enzyme to reach the highest possible yield of **6**.

### Kinetic analysis of the synthesis reaction using the glycosynthase BgaC/Glu233Gly

Kinetic analysis of BgaC/Glu233Gly revealed an apparent K_M,app_ of 0.22 mm and a v_max_ of 2.6 mU/mg regarding the donor substrate αGalF **2** (Fig. 2). For comparison, a K_M_-value of 1.0 mm was reported for **2** using *p*NP-αGalNAc as the acceptor substrate [31]. Interestingly, K_M,app_ and v_max_ could not be determined for the novel acceptor substrate **1** as the fitted curve did not show saturation within the investigated substrate concentration range. In a previous study, K_M_ values between 0.71 mm and 0.41 mm were reported for the aryl-glycosides *p*NP-αGlcNAc and *p*NP-αGalNAc, respectively [31]. The difference in K_M_ values may be due to a generally higher affinity of the wild type glycosidase for aryl-substituted glycosides compared to unmodified acceptor substrates [38]. It can be assumed that the same holds true for the respective glycosynthase.

### Synthesis of TF-antigen-NGPs (**12**)

Variation of the ratio of propargyl-EG1-NHS-ester **9** and available amines of BSA **8** results in variable alkynyl-modification of BSA. This is reflected by the analysis of the coupling reaction of **9** with BSA **8** via TNBSA-assay and SDS-PAGE. A higher number than 60 modified residues per mol BSA are obtained when molar excesses of more than 4:1 of are applied. While the results of the TNBSA-assay show that all 60 lysine residues of BSA react with **9**, the apparent molecular weights in SDS-PAGE analysis indicate that even more than 60 molecules of **6** are attached to the protein when higher excesses of **9** are used. The TNBSA reagent is known to react exclusively with the ε-amines of lysine residues [39] and is therefore limited to the quantification of available lysine residues in the BSA sequence. In contrast, SDS-PAGE detects any molecular weight shift independent from the site at which the modification occurred. We hypothesize that **9** reacts with additional sites in the BSA structure apart from the lysine residues. Previous studies report the formation of ester bonds with serine, tyrosine, and threonine residues of proteins and small peptides [40, 41]. These residues constitute 13.5% of the BSA sequence. The formation of ester linkages is further enhanced if a histidine residue is located in direct proximity to one of these side chains [42]. The histidine-catalyzed *O*-acylation of side chains containing OH-groups is increased at lower pH-values between pH 6.7–7.8 as the low pKs-value of His-side chains causes the imidazole ring to be deprotonated. This, in turn, results in increased nucleophilicity and thus higher reactivity towards NHS-activated esters. The *N*-acylated imidazole ring serves as an intermediate in the reaction and the alkynyl-crosslinker is subsequently transferred onto directly adjacent hydroxyl-bearing side chains. This mechanism has been applied before in the selective targeting of tyrosine residues of bovine α-lactalbumin [43]. Moreover, it can be expected that the side chains of a globular protein are subject to additional intramolecular interaction (e.g. hydrogen bonding) which may have an effect on their nucleophilicity and thus can potentially alter their reactivity towards acylation. Therefore, it is important to put into consideration that at high excess ratios of **9** additional sites of BSA **8** (Ser, Thr or Tyr) are probably modified. These modifications are not detected with the TNBSA assay. For lower ratios, the results obtained via the TNBSA assay and SDS-PAGE are in accordance and yield comparable numbers of modified side chains (Table 1). We assume that the lysine residues are acetylated at lower NHS-ester excess ratios.

Our data demonstrate that the number of attached glycans can be controlled by the number of alkynyl-functionalized amino acid residues and a constant azide/alkyne ratio. The conversion of the *click* reactions with respect to the number of alkynyl residues are constant between 42 and 51%, if the number of alkynyl residues per BSA is at least 33 or higher (Table 1). This is achieved by using a propargyl-EG1-NHS ester **9**/terminal amine-ratio of at least 1:1 or higher. The moderate yields may be due to partially inaccessible alkynyl-carrying side chains for the TF-antigen-azide **6**. However, previous studies on CuAAC-catalyzed labeling of human serum albumin suggest that a higher excess of the glycan during CuAAC might further increase the glycosylation degree to 60 glycans/BSA [44]. Our results suggest that with propargyl-EG1-NHS ester **9** even glycan densities beyond 60 residues per BSA seem possible due to the *O*-acylation of Tyr, Thr and Ser within the BSA sequence.

To sum up, our approach enables the facile formation of neo-glycoproteins combining enzymatic and chemical protocols. Since the availability of **6** is the limiting factor in terms of costs and effort, we attempted to use a reasonable excess of the disaccharide during CuAAC, resulting in conversions of around 50%. The resulting neo-glycoproteins contained between 2 and 53 glycans per molecule. Therefore, the presented protocol enables the formation of tailor-made neo-glycoproteins with a desired glycan density.

### Binding of Gal-3 to TF-antigen NGPs (**12**)

Our results show that binding values of Gal-3 to immobilized NGPs displaying different numbers of glycans increased with increasing glycan density (Fig. 5). However, in comparison to ASF, the standard for Gal-3 binding analysis, binding affinity remained low even at high occupation numbers. In addition, an effect of multivalent presentation of the TF-antigen was not observed (Fig. 6). In contrast, inhibition studies showed that multivalent presentation of the TF-antigen on BSA neoglycoproteins enhances the relative inhibitory potency by two orders of magnitude. The effect of multivalency, however, was dependent on the degree of glycan decoration. BSA10, presenting 10 TF-antigen glycans, exhibits a 142-fold higher relative inhibitory potency (RIP) compared to monovalent TF-antigen. NGPs with a higher glycan density both showed lower RIP-values (98 for BSA37 and 92 for BSA46). The RIP value relative to the glycan density reveals that multivalency effects occurred most strongly for BSA10 (RIP/glycan of 14.2). Apparently, decoration with only 10 glycans/BSA triggers a stronger glyco-cluster effect [45] than a dense decoration with 37 (RIP/glycan of 2.6) or 46 (RIP/glycan of 2) glycans, respectively. In comparison to our results depicted in Fig. 5, TF-antigen NGPs are readily accessible for binding Gal-3 in solution whereas immobilization of TF-antigen NGPs reduces binding of Gal-3. These results are in accordance with a previous study by Stowell et al., who found that no significant binding was detected when the TF-antigen was immobilized on a glycan-array [46].

Previous studies indicate that the monovalent attachment of the TF-antigen to a MUC1-like peptide backbone via an α1-linkage increases the affinity towards Gal-3 relative to the unconjugated glycan approximately 10-fold [20, 47]. In addition, the multivalent display of the TF-antigen on natural antifreeze-proteins from cod leads to a significant glyco-cluster effect with K_D_-values approximately 6 orders of magnitude lower than for the unconjugated TF-disaccharide [22]. In contrast, multivalent presentation of the TF-antigen on glycan-arrays without linkage to a peptide backbone did not result in a stronger binding [47]. Taken together, the coupling of the glycan to its natural MUC1-scaffold or a similar peptide sequence might be important for increasing its affinity towards Gal-3. Gal-3 features a binding groove with the subsites A-E. Subsite C and D bind the disaccharides lactose (Gal(β1,4)Glc) or LacNAc type 2 (Gal(β1,4)GlcNAc), whereas subsite A and B present a more flexible binding pocket for additional interactions with e.g. poly-LacNAc glycan chains [48–50]. We demonstrated sub-nanomolar binding constants of the BSA-coupled tetrasaccharide LacdiNAc-LacNAc due to glycan binding to the subsites A-E of Gal-3 with additional binding interaction of the terminal GalNAc residues in subsite A [17]. Therefore, BSA as a protein scaffold for glycan presentation can be regarded as functional for Gal-3 binding.

Monovalent disaccharides generally exhibit lower binding affinities for Gal-3 when compared to poly-LacNAc. LacNAc type 2 is the best disaccharide ligand in comparison to LacNAc type 1 (Gal(β1,3)GlcNAc) and the TF-antigen [21, 48]. The TF-antigen disaccharide differs from LacNAc type 2 with respect to the reducing sugar being GalNAc instead of GlcNAc as well as the terminal Gal being linked via a β1,3-linkage instead of a β1,4-linkage. Structural analysis of Gal-3 binding the TF-antigen disaccharide revealed that the GalNAc-moiety is responsible for a unique binding pattern based on a Glu165-water-Arg186-water-motif [8]. This binding pattern is not observed with other glycan ligands of Gal-3. Binding of the Gal-moiety at subsite C includes interaction with the same amino acids as described for binding of LacNAc type 2 [8, 48]. Recently reported binding constants of Gal-3 for TF-antigen (K_D_ 47 μm) [8], LacNAc type 2 (K_D_ 33 μm) and LacNAc type 1 (K_D_ 93 μm) [51] suggest subtle differences. When comparing LacNAc type 1 and the TF-antigen, exchange of GlcNAc with GalNAc in combination with the β1,3-linkage apparently result in slightly better binding.

## Conclusions

We here demonstrate the efficient enzymatic synthesis of the EG3-azide-functionalized TF-antigen **3** and its coupling to BSA via conventional *click* chemistry. The glycan density of the resulting neo-glycoproteins is controllable by the amount of propargyl-EG1-NHS ester **4** used during alkynyl-functionalization of the protein. While monovalent TF-antigen has proven to be a weak ligand for Gal-3, the presented data shows that multivalent glycan presentation does not lead to a higher binding affinity as previously demonstrated for immobilized LacNAc-based neo-glycoproteins. This may be due to differences in the glycan presentation on BSA and the Gal-3 binding mode for the TF-antigen. In contrast, the inhibitory potency of TF-antigen NGPs for Gal-3 binding to ASF in solution was improved by two orders of magnitude. The NGP with 10 TF-antigen glycans depicted the highest relative inhibitory potential per glycan, indicating that a minimal glycan density of NGPs is favorable for inhibiting binding of Gal-3 to the glycoprotein ASF.

## Electronic supplementary material

ESM 1 (PDF 547 kb)
