# Supplementary material for: Synthesis of the Thomsen-Friedenreich-antigen (TF-antigen) and binding of Galectin-3 to TF-antigen presenting neo-glycoproteins
Source: Glycoconj J. 2020 May 4;37(4):457–70. doi: 10.1007/s10719-020-09926-y (PMC7329766; doi:10.1007/s10719-020-09926-y)
Supplement: Supplementary file 1 — (PDF 547 kb) [file 10719_2020_9926_MOESM1_ESM.pdf]

Supporting Information

Synthesis of the Thomsen-Friedenreich-Antigen (TF-antigen) and binding of Galectin-3 to TF-antigen presenting neo-glycoproteins

Marius Hoffmann,[a] Marc R. Hayes,[b] Jörg Pietruszka[b,c] and Lothar Elling [a,*]

[a] Laboratory for Biomaterials, Institute for Biotechnology and Helmholtz-Institute for Biomedical Engineering, RWTH Aachen University, Pauwelsstraße. 20, 52074 Aachen, Germany

[b] Institute for Bioorganic Chemistry, Heinrich Heine University Düsseldorf at Forschungszentrum Jülich, 52426 Jülich, Germany

[c] Forschungszentrum Jülich, IBG-1: Biotechnology, 52426 Jülich, Germany

*corresponding author

Marius Hoffmann
m.hoffmann@biotec.rwth-aachen.de
ORCID 0000-0001-6727-6827

Marc R. Hayes
m.hayes@fz-juelich.de
ORCID 0000-0003-0468-2398

Jörg Pietruszka
j.pietruszka@fz juelich.de
ORCID 0000-0002-9819-889X

Lothar Elling
L.Elling@biotec.rwth-aachen.de
ORCID 0000-0002-3654-0397

Synthesis of TF-antigen-PEG3-azide 3

Fig. S1 shows that the donor substrate *p*NP-Gal is fully converted after approx. 15 min. The concentration of the desired product Galβ1,3GalNAcα1-EG3-azide (TF-antigen-EG3-azide, **3**) increases and has its highest value after 8 min (2.9 mM).

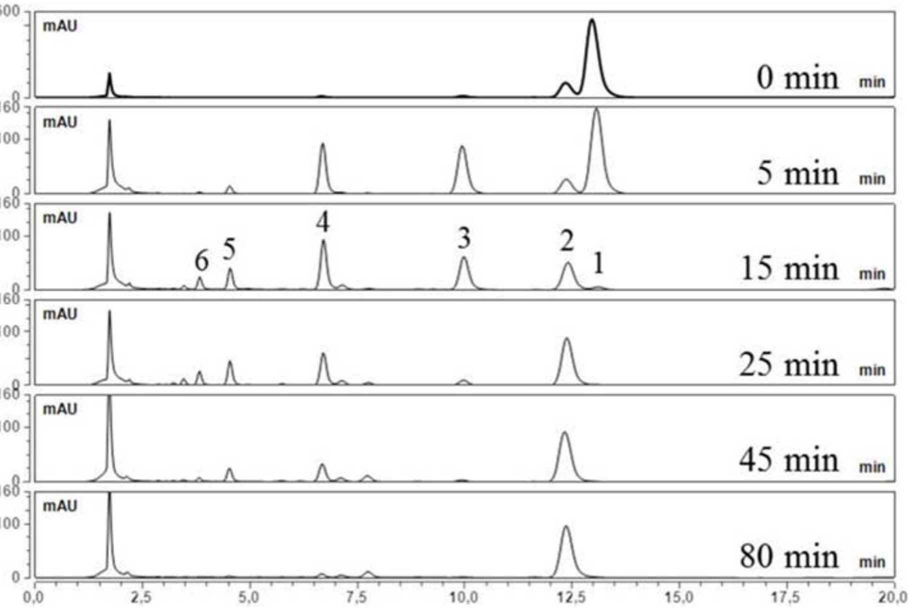

Fig. S1 HPLC-overlay of the synthesis of Galβ1,3GalNAcα1-PEG3-azide **6** using BgaC. The numbers indicate substrate and product peaks in the reaction. 1: *p*NP-Gal; 2: GalNAcα1-EG3-azide **1**; 3: Galβ1,3GalNAcα1-PEG3-azide **3**; 4: GalβGalβ1,3GalNAcα1-EG3-azide; 5: GalβGalβ1,3Galβ1,3GalNAcα1-EG3-azide; 6: GalβGalβGalβGalβ1,3GalNAcα1-PEG3-azide. *p*NP elutes after 25 min and is therefore not included in this chromatogram. Peak numbers 2, 3 and 4 were isolated and identified *via* LC/ESI-MS.

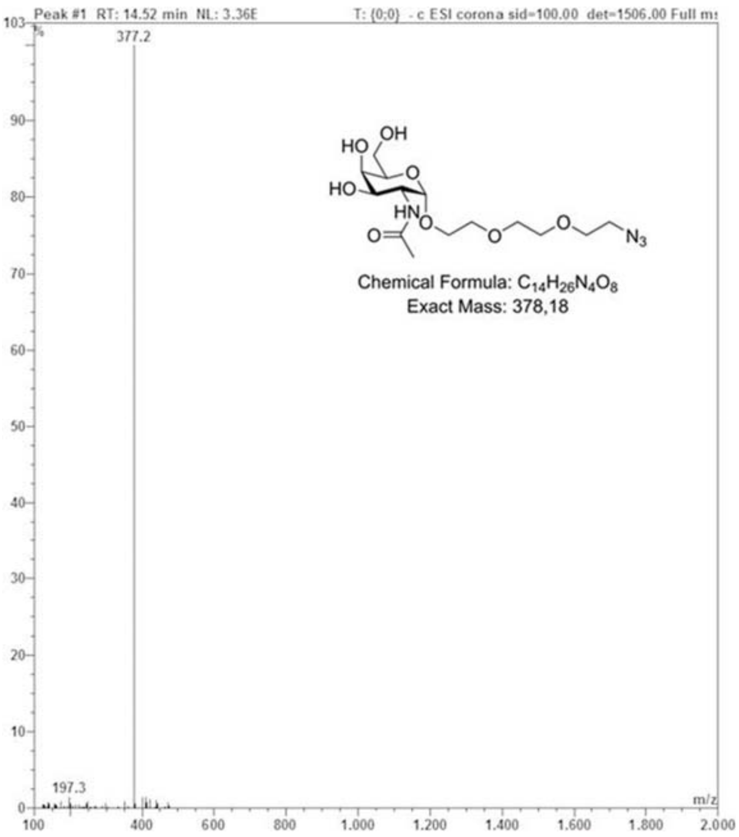

Fig. S2 Mass spectrum of the standard GalNAcα1-EG3-azide **1** (Peak 2 in Fig. S1). The measurement was conducted *via* ESI-MS (Finnigan Surveyor MSQ Plus, Thermo Scientific, needle voltage = 4 kV, temperature = 400 °C, cone voltage = 100 V, negative mode). The respective $[M-H]^-$ ion at *m/z* 377.2 corresponds to the calculated mass of [M] 378.18 g/mol.

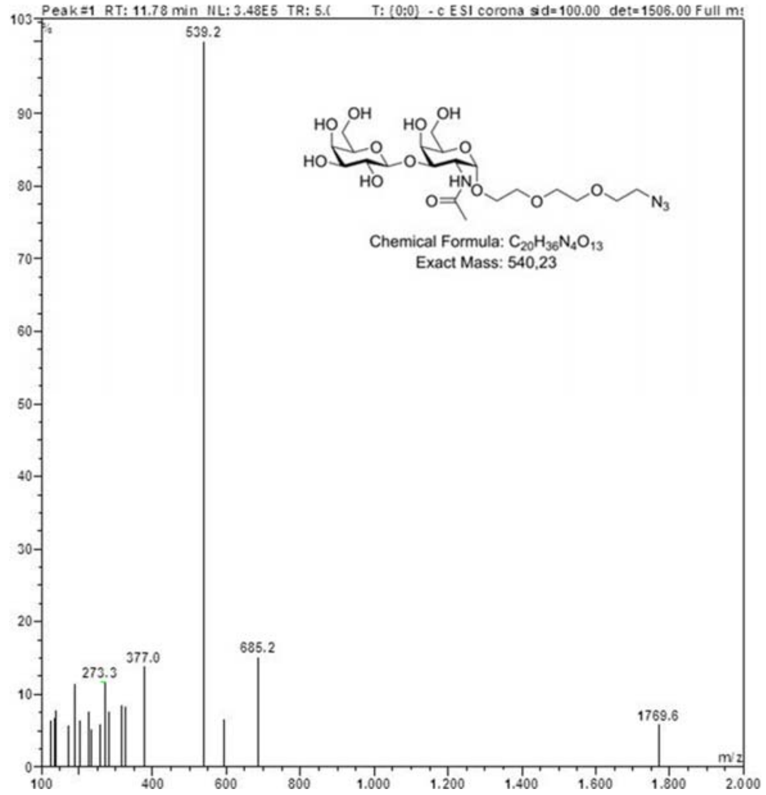

Fig. S3 Mass spectrum of the reaction product Galβ1,3GalNAcα-1-EG3-azide **3** using BgaC glycosidase (Peak 3 in Fig. S1). The measurement was conducted *via* ESI-MS (Finnigan Surveyor MSQ Plus, Thermo Scientific, needle voltage = 4 kV, temperature = 400 °C, cone voltage = 100 V, negative mode). The respective $[M-H]^-$ ion at *m/z* 539.2 corresponds to the calculated mass of [M] 540.2 g/mol.

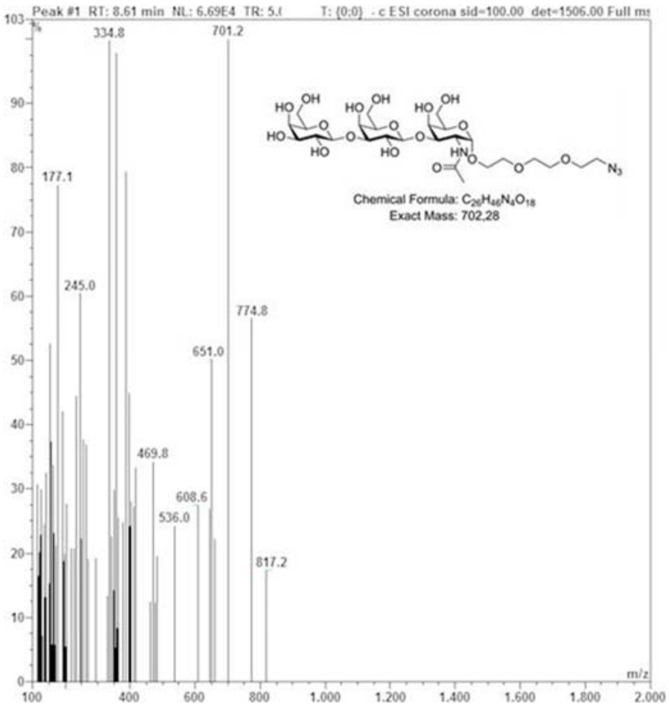

Fig. S4 Mass spectrum of the reaction product Galβ1,3Galβ1,3GalNAcα1-EG3-azide using BgaC glycosidase (Peak 4 in Fig. S1). The measurement was conducted *via* ESI-MS (Finnigan Surveyor MSQ Plus, Thermo Scientific, needle voltage = 4 kV, temperature = 400 °C, cone voltage = 100 V, negative mode). The respective $[M\text{-}H]^-$ ion at *m/z* 701.2 corresponds to the calculated mass of [M] 702.3 g/mol.

Kinetic analysis of BgaC/Glu233Gly

Equation S1 Michaelis-Menten Equation

$$v = \frac{v_{max} * [S]}{K_M + [S]}$$

V:	Reaction velocity [U/mg]
v_{max}:	maximal reaction velocity [U/mg]
[S]:	Substrate concentration [mM]
K_M:	Affinity constant [mM]

Equation S2 Hill Equation

$$v = \frac{v_{max} * [S]^n}{[K_M]^n + [S]^n}$$

V:	Reaction velocity [U/mg]
v_{max}:	maximal reaction velocity [U/mg]
[S]:	Substrate concentration [mM]
K_M:	Affinity constant [mM]
n:	Hill coefficient [-]

TF-antigen-PEG3-azide synthesis using BgaC/Glu233Gly galactosynthase

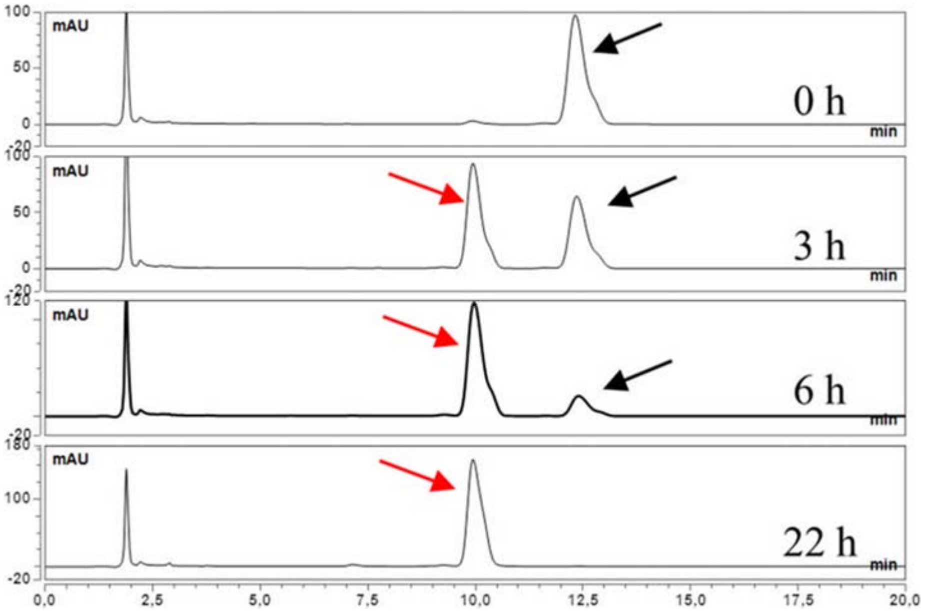

Fig. S5 HPLC-overlay of the synthesis of Galβ1,3GalNAcα-1-EG3-azide **6** using BgaC/Glu233Gly. Red arrows indicate the growing product peak. Black arrows indicate the substrate peak.

Product characterization

Mass spectrometry – LC/ESI-MS

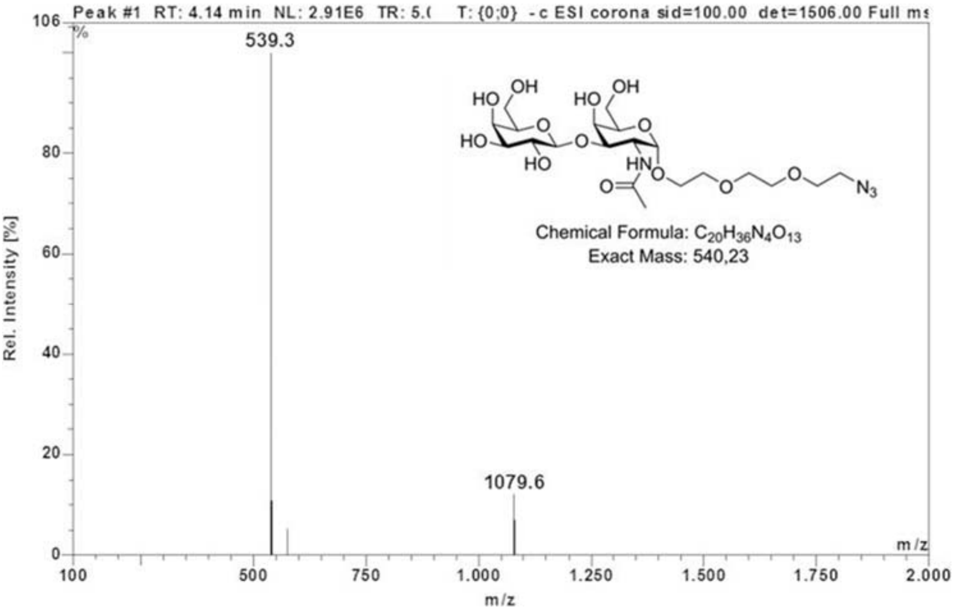

Fig. S6 Mass spectrum of the reaction product Galβ1,3GalNAcα1-EG3-azide **6** using BgaC/Glu233Gly. The measurement was conducted *via* ESI-MS (Finnigan Surveyor MSQ Plus, Thermo Scientific, needle voltage = 4 kV, temperature = 400 °C, cone voltage = 100 V, negative mode). The respective $[M-H]^-$ ion at *m/z* 539.3 corresponds to the calculated mass of [M] 540.23 g/mol.

NMR analysis

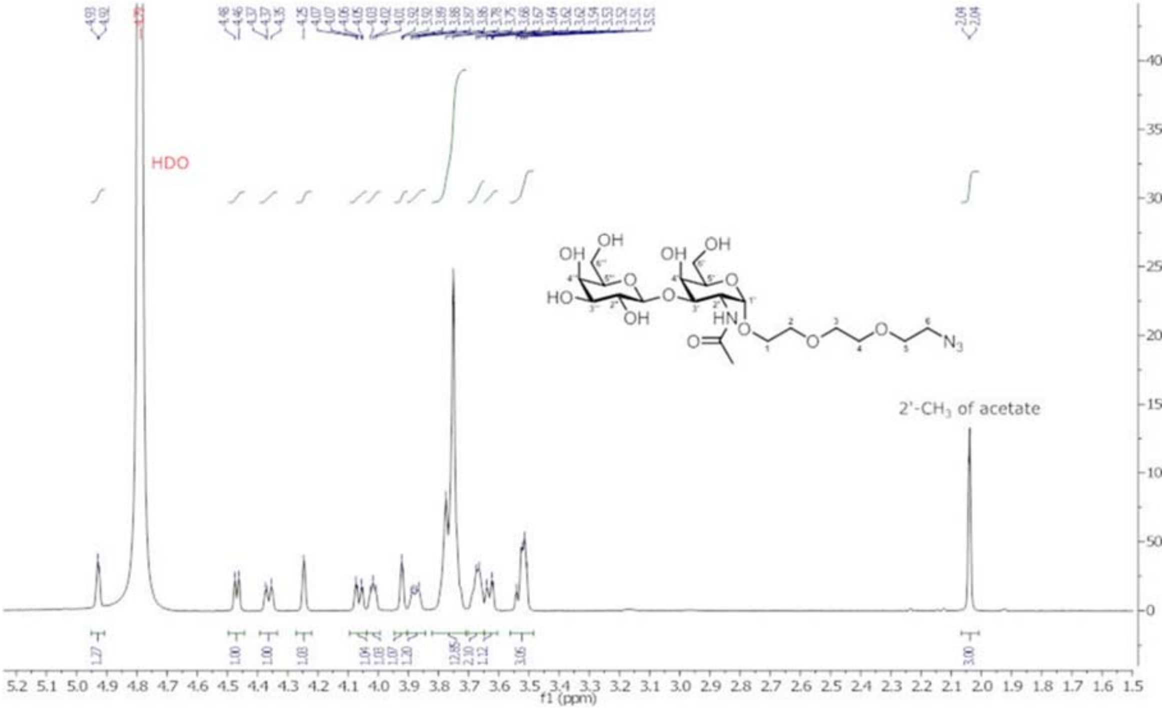

Fig. S7 ^{1}H-NMR analysis of the reaction product Galβ1,3GalNAcα1-EG3-azide **6** using BgaC/Glu233Gly recorded in D_2O.

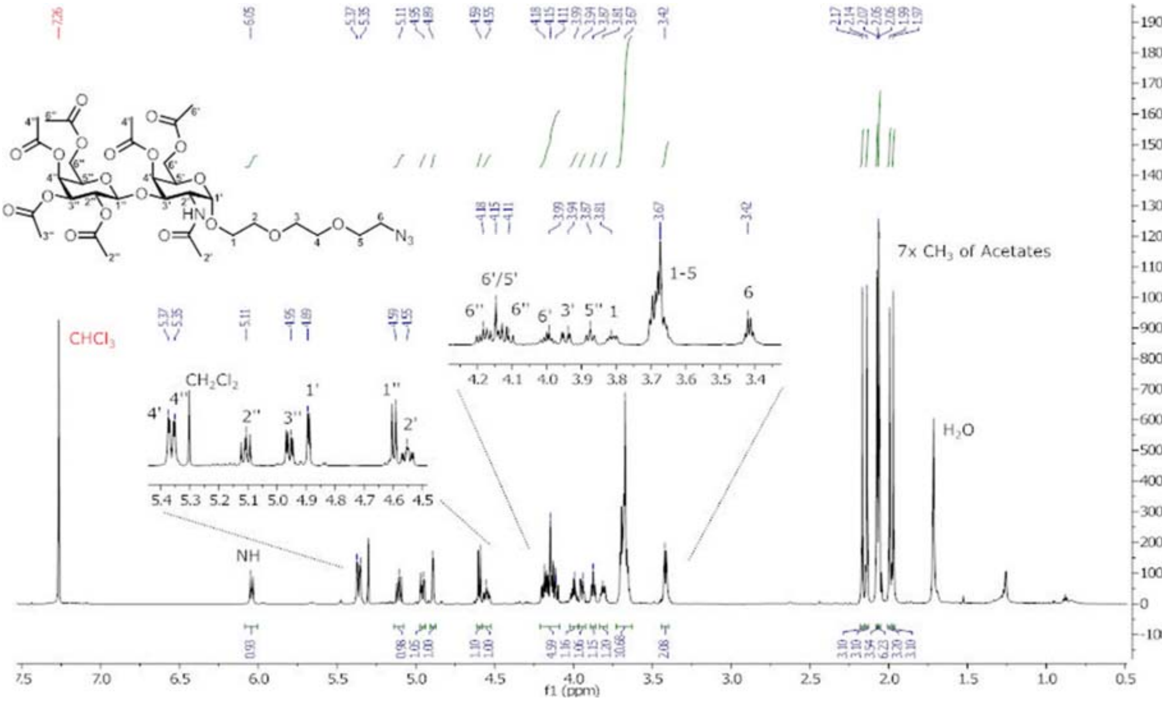

Fig. S8 ^{1}H-NMR analysis of 8-azido-3,6-dioxaoctyl-2-acetamido-4,6-di-*O*-acetyl-2-deoxy-3-*O*-(2,3,4,6-tetra-*O*-acetyl-β-D-galactopyranosyl)-α-D-galactopyranoside recorded in $CDCl_3$.

^{1}H-NMR ($CDCl_3$, 600 MHz)

δ [ppm] = 6.04 (d, $^3J_{NH,2'}$ = 9.5 Hz, 1 H, N*H*), 5.37 (d, $^3J_{4'',3''}$ = 3.1 Hz, 1 H, 4′-H), 5.35 (dd, $^3J_{4'',3''}$ = 3.3 Hz, $^3J_{4'',5''}$ = 1.1 Hz, 1 H, 4″-H), 5.10 (dd, $^3J_{2'',3''}$ = 10.4 Hz, $^3J_{2'',1''}$ = 7.9 Hz, 1 H, 2″-H), 4.95 (dd, $^3J_{3'',2''}$ = 10.4 Hz, $^3J_{3'',4''}$ = 3.3 Hz, 1 H, 3″-H), 4.89 (d, $^3J_{1',2'}$ = 3.6 Hz, 1 H, 1′-H), 4.59 (d, $^3J_{1'',2''}$ = 7.9 Hz, 1 H, 1″-H), 4.55 (ddd, $^3J_{2',1'}$ = 3.6 Hz, $^3J_{2',3'}$ = 10.9 Hz, $^3J_{2',NH}$ = 9.5 Hz, 1 H, 2′-H), 4.18 (dd, $^3J_{6a',6b'}$ = 10.3 Hz, $^3J_{6a',6b'}$ = 6.0 Hz, 1 H, 6′$_{a/b}$-H), 4.17-4.12 (m, 2 H, 5′-H, 6″$_{a/b}$-H), 4,11 (dd, $^3J_{6b',6a'}$ = 10.3 Hz, $^3J_{6b',6a'}$ = 7.5 Hz, 1 H, 6′$_{a/b}$-H), 3.99 (m$_c$, 1 H, 6″$_{a/b}$-H), 3.94 (dd, $^3J_{3',2'}$ = 10.9 Hz, $^3J_{3',4'}$ = 3,1 Hz, 1 H, 3′-H), 3.87 (ddd, $^3J_{5'',4''}$ = 1.1 Hz, $^3J_{5'',6a/b''}$ = 7.3 Hz, $^3J_{5'',6a/b''}$ = 6.2 Hz, 1 H, 5″-H), 3.81 (ddd, $^3J_{1a,1b}$ = 10.5 Hz, $^3J_{1a,2a}$ = 2.6 Hz, $^3J_{1a,2b}$ = 5.1 Hz, 1 H, 1$_a$-H), 3.67 (m, 9 H, 1$_b$-H, 2-H, 3-H, 4-H, 5-H), 3.42 (t, $^3J_{6,5}$ = 4.6 Hz, 1 H, C*H*$_2$N$_3$), 3.41 (t, $^3J_{6,5}$ = 4.6 Hz, 1 H, C*H*$_2$N$_3$), 2.16 (s, 3 H, *CH*$_3$-3″), 2.13 (s, 3 H, *CH*$_3$-4′), 2.07 (s, 3 H, *CH*$_3$-2″), 2.06 (s, 3 H, *CH*$_3$-6′/6″), 2.06 (s, 3 H, *CH*$_3$-4″), 1.99 (s, 3 H, *CH*$_3$-2′), 1.97 (s, 3 H, *CH*$_3$-6′/6″).

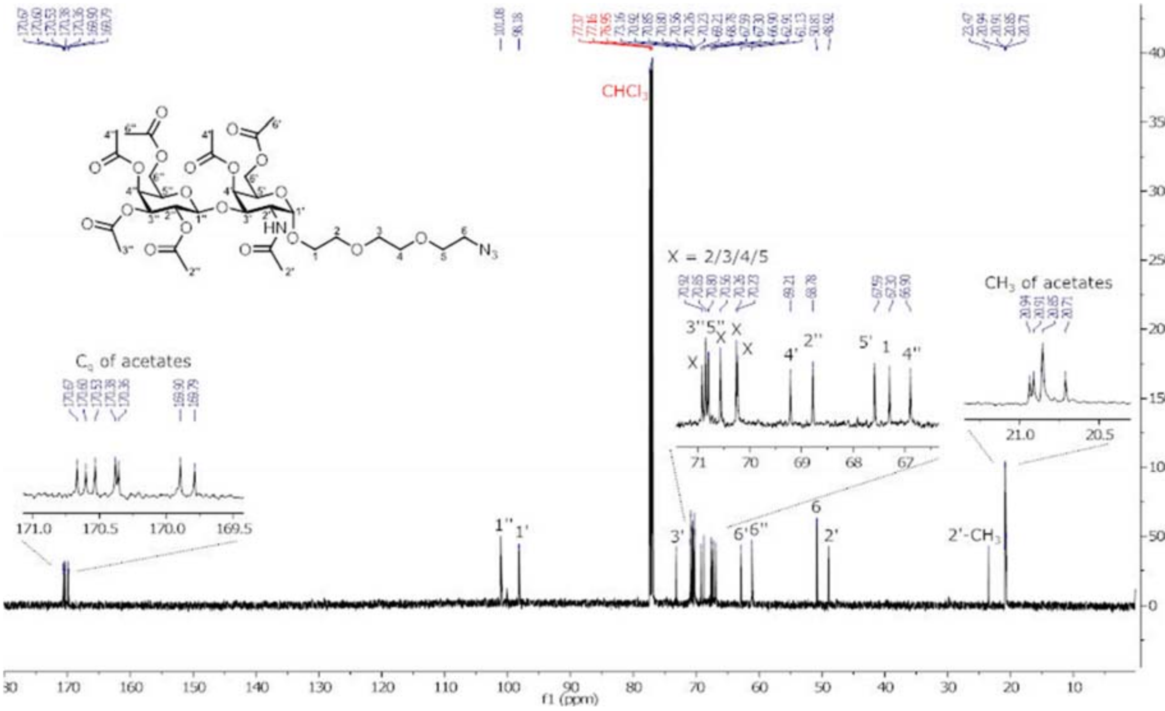

Fig. S9 ^{13}C-NMR of 8-azido-3,6-dioxaoctyl-2-acetamido-4,6-di-*O*-acetyl-2-deoxy-3-*O*-(2,3,4,6-tetra-*O*-acetyl-β-D-galactopyranosyl)-α-D-galactopyranoside recorded in $CDCl_3$.

^{13}C-NMR ($CDCl_3$, 151 MHz)

δ [ppm] = 170.67 (C$_q$-6), 170.60 (C$_q$-4″), 170.53 (C$_q$-3″), 170.38 (C$_q$-4′), 170.36 (C$_q$-6), 169.90 (C$_q$-2), 169.79 (C$_q$-2″), 101.08 (C-1″), 98.18 (C-1′), 73.16 (C-3′), 70.92, 70.85 (PEG, 2 C, C-2/3/4/5), 70.80 (C-3″), 70.56 (C-5″), 70.26, 70.23 (PEG, 2 C, C-2/3/4/5), 69.21 (C-4′), 68.78 (C-2″), 67.59 (C-5′), 67.30 (C-1), 66.90 (C-4″), 62.91 (C-6′), 61.13 (C-6″), 50.81 (C-6), 48.92 (C-2′), 23.47 (CH_3-2′), 20.94 (CH_3-4″), 20.91, 20.85, 20.71 (5 CH_3-2″, -4′, -3″, -6′, -6″).

Synthesis of neo-glycoproteins

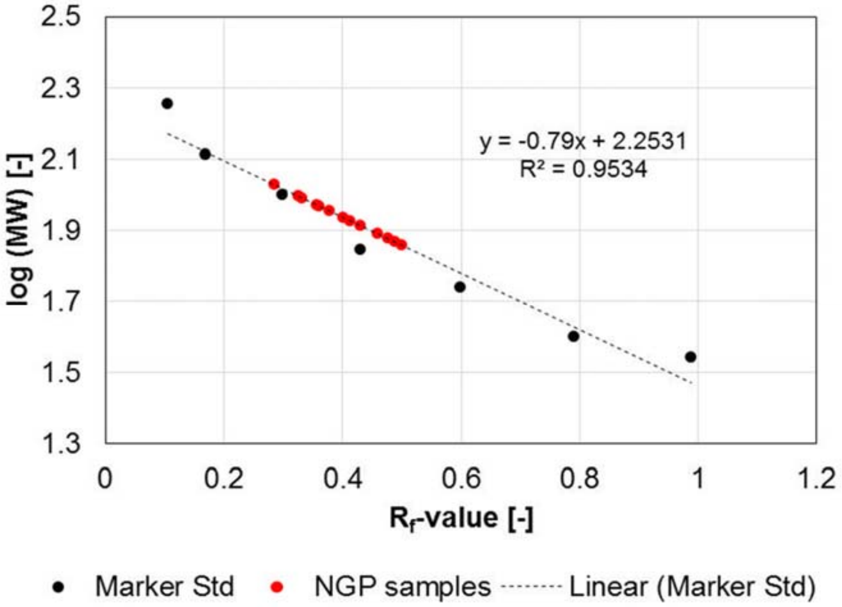

Fig. S10 Linear regression for the calculation of the number of glycans per BSA after coupling of the TF-antigen-azide **6** *via* CuAAC. Marker Std: PageRuler Prestained Protein Ladder (ThermoFisher, Waltham, USA). The migrated distance of each molecular weight band in the marker and the sample was measured and the R_f-value was calculated. Molecular weight of the samples was calculated by using linear regression of the standard protein ladder. The resulting values were used for the calculation of the molecular weight shift between modified and unmodified BSA.

Table S1 Number of alkynylated and glycosylated sites in the BSA protein sequence according to analysis *via* TNBSA assay and SDS-PAGE after synthesis on a 6 mL scale. Yields of the *click* reaction were calculated on the basis of the number of alkynes present according to the TNBSA assay (for NHS-ester **9** molar excess of 2) or SDS-PAGE (for NHS-ester **9** molar excess 10 and 20), respectively. The TNBSA-assay was conducted in triplicates. The standard deviation of the mean is provided behind the calculated number of alkynyl groups.

NHS-ester/ terminal amine [mol/mol]	Alkynes/BSA (TNBSA) [mol/mol]	Alkynes/BSA (PAGE) [mol/mol]	TF-Ag/BSA (PAGE) [mol/mol]	Yield CuAAC [%]
2	51 ± 0.2	40	10	51
10	60 ± 0.2	82	37	45
20	60 ± 0.3	110	46	42

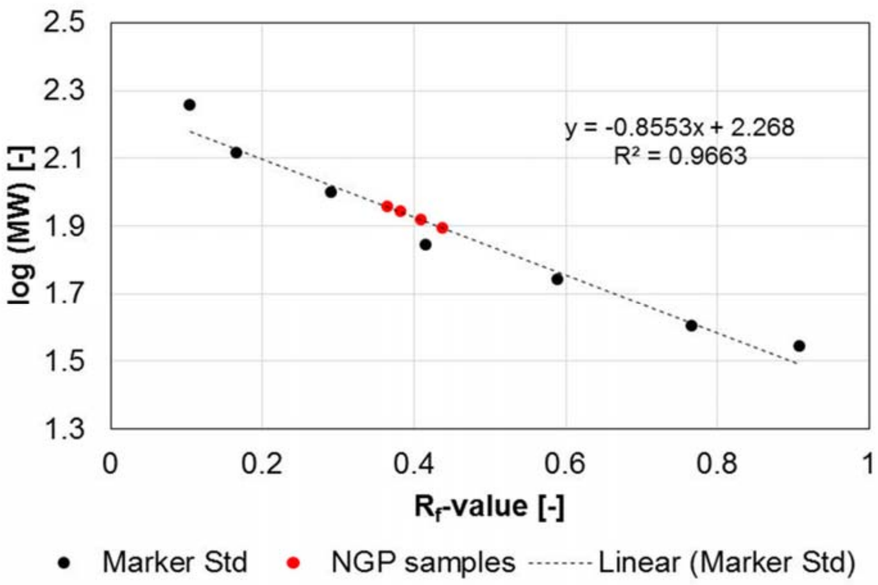

Fig. S11 Linear regression for the calculation of the number of glycans per BSA after coupling of the TF-antigen-azide **6** *via* CuAAC using a volume of 6 mL. Marker Std: PageRuler Prestained Protein Ladder (ThermoFisher, Waltham, USA). The migrated distance of each molecular weight band in the marker and the sample was measured and the R_f-value was calculated. Molecular weight of the samples was calculated by using linear regression of the standard protein ladder. The resulting values were used for the calculation of the molecular weight shift between modified and unmodified BSA
